# Fine Mapping of a Dravet Syndrome Modifier Locus on Mouse Chromosome 5 and Candidate Gene Analysis by RNA-Seq

**DOI:** 10.1371/journal.pgen.1006398

**Published:** 2016-10-21

**Authors:** Nicole A. Hawkins, Nicole J. Zachwieja, Alison R. Miller, Lyndsey L. Anderson, Jennifer A. Kearney

**Affiliations:** 1 Department of Pharmacology Northwestern University Feinberg School of Medicine Chicago, Illinois, United States of America; 2 Department of Medicine, Vanderbilt University, Nashville, Tennessee, United States of America; Columbia University Medical Center, UNITED STATES

## Abstract

A substantial number of mutations have been identified in voltage-gated sodium channel genes that result in various forms of human epilepsy. *SCN1A* mutations result in a spectrum of severity ranging from mild febrile seizures to Dravet syndrome, an infant-onset epileptic encephalopathy. Dravet syndrome patients experience multiple seizures types that are often refractory to treatment, developmental delays, and elevated risk for SUDEP. The same sodium channel mutation can produce epilepsy phenotypes of varying clinical severity. This suggests that other factors, including genetic, modify the primary mutation and change disease severity. Mouse models provide a useful tool in studying the genetic basis of epilepsy. The mouse strain background can alter phenotype severity, supporting a contribution of genetic modifiers in epilepsy. The *Scn1a*^*+/-*^ mouse model has a strain-dependent epilepsy phenotype. *Scn1a*^*+/-*^ mice on the 129S6/SvEvTac (129) strain have a normal phenotype and lifespan, while [129xC57BL/6J]F1-*Scn1a*^*+/-*^ mice experience spontaneous seizures, hyperthermia-induced seizures and high rates of premature death. We hypothesize the phenotypic differences are due to strain-specific genetic modifiers that influence expressivity of the *Scn1a*^*+/-*^ phenotype. Low resolution mapping of *Scn1a*^*+/-*^ identified several Dravet syndrome modifier (*Dsm*) loci responsible for the strain-dependent difference in survival. One locus of interest, *Dsm1* located on chromosome 5, was fine mapped to a 9 Mb region using interval specific congenics. RNA-Seq was then utilized to identify candidate modifier genes within this narrowed region. Three genes with significant total gene expression differences between 129S6/SvEvTac and [129xC57BL/6J]F1 were identified, including the GABA_A_ receptor subunit, *Gabra2*. Further analysis of *Gabra2* demonstrated allele-specific expression. Pharmological manipulation by clobazam, a common anticonvulsant with preferential affinity for the GABRA2 receptor, revealed dose-dependent protection against hyperthermia-induced seizures in *Scn1a*^*+/-*^ mice. These findings support *Gabra2* as a genetic modifier of the *Scn1a*^*+/-*^ mouse model of Dravet syndrome.

## Author Summary

Epilepsy is a neurological disorder affecting approximately 3 million Americans and 1% of the worldwide population. Approximately 70% of patients diagnosed with epilepsy have a genetic basis for their disease. The same genetic mutation can result in epilepsy with varying clinical severity in some individuals. This suggests that additional factors modify the effect of the primary mutation, resulting in the differences observed. Mouse models of epilepsy also exhibit variable severity depending on the inbred strain background. This is a useful model system that enables us to determine other genetic factors that influence phenotype presentation. This study investigates a mouse model of Dravet syndrome. Similar to Dravet syndrome patients, the mice experience a variety of seizure types and have increased risk for premature death. Interestingly, the Dravet syndrome phenotype is completely masked on one mouse strain background. Through a series of genetic and pharmacological techniques we were able to identify a gene that is likely responsible for the strain-dependent phenotype difference of Dravet mice. Identifying genes that modify diseases can help predict patient outcomes and suggest new therapies for the treatment of epilepsy.

## Introduction

Epilepsy is one of the most common neurological disorders, affecting approximately 50 million people worldwide. Approximately two-thirds of human epilepsies are presumed to have a genetic basis. Voltage-gated sodium channel gene mutations are the most common cause of monogenic epilepsy. Mutations in *SCN1A*, encoding Nav1.1, result in a broad spectrum of disorders ranging from simple febrile seizures to Dravet syndrome, a severe, infant-onset epileptic encephalopathy with devastating outcomes [1]. Dravet syndrome patients have multiple seizures types that are refractory to treatment, as well as delays of psychomotor and cognitive development and a high risk of sudden unexpected death in epilepsy (SUDEP) [2].

Within genetic epilepsies, diverse phenotypes often result from identical sodium channel mutations [3]. This suggests that disease severity is influenced by other factors, including genetic modifiers. The genetic basis of epilepsy can be studied using mouse models that recapitulate hallmark features of human epilepsies. Frequently, mutations that result in seizures show strain-dependent phenotype variability, suggesting a contribution of genetic modifiers in epilepsy [4]. Heterozygous deletion of mouse *Scn1a* models features of Dravet syndrome, including spontaneous seizures, thermal seizure sensitivity, cognitive deficits, and increased mortality [5,6]. Interestingly, expressivity of the *Scn1a*^+/-^ phenotype is highly strain-dependent. When the *Scn1a*^+/-^ mutation is maintained on the 129S6/SvEvTac (129) strain, 129.*Scn1a*^+/-^ mice have no overt phenotype and a normal lifespan. When 129.*Scn1a*^+/-^ mice are crossed with C57BL/6J (B6), the resulting [129xB6]F1.*Scn1a*^+/-^ (F1.*Scn1a*^+/^) offspring exhibit spontaneous seizures and 75% mortality by 8 weeks of age [7]. We previously mapped several Dravet syndrome modifier (*Dsm*) loci that influence the strain-dependent difference in survival [7].

In the current study, we used interval-specific congenic (ISC) strains to confirm and fine map the *Dsm1* locus on chromosome 5. We then performed candidate gene analysis using an RNA-Seq approach, and identified *Gabra2* as a high priority candidate gene. Further evaluation of *Gabra2* by expression analysis and pharmacological manipulation support *Gabra2* as a putative modifier gene that influences survival in the *Scn1a*^*+/-*^ Dravet model.

## Results

### Fine Mapping of Dsm1 with Interval Specific Congenic Strains

**Fig 1:**
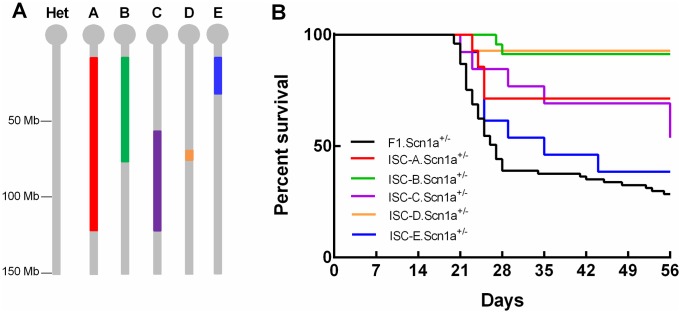
Fine mapping of *Dsm1* with ISC strains. A. *Dsm1* ISC lines A-E carry varying 129 derived chromosome 5 segments (colors) on a congenic B6 background (grey). B. Survival of resulting F1.KO offspring was monitored to 8 weeks of age. Kaplan Meier survival plot is shown. Survival was significantly improved in mice with homozygous 129/129 alleles in ISC-A (p<0.01), ISC-B (p<0.0001), ISC-C (p<0.05), and ISC-D (p < 0.0002) compared to F1.KO heterozygous controls. P values were determined by LogRank Mantel-Cox test.

Inheritance of B6 alleles in *Dsm1* increased the risk of early death in *Scn1a*^+/-^ mice [7]. Low resolution mapping identified the *Dsm1* 1.5 LOD support interval as 27.3–41.3 cM on chromosome 5, with a peak at 34 cM [7]. To refine the *Dsm1* map interval, five interval specific congenic (ISC) lines were generated with varying 129-derived chromosome 5 segments within *Dsm1* on a congenic B6 background (Fig 1A). Each ISC line was crossed with 129.*Scn1a*^+/-^ to generate F1.*Scn1a*^+/-^offspring carrying either homozygous (129/129) or control heterozygous (129/B6) alleles in *Dsm1*. Survival of F1.*Scn1a*^+/-^ mice was then monitored until 8 weeks of age (Fig 1B).

A significant improvement in survival was observed with lines ISC-A (4–118 Mb; p<0.01), ISC-B (4–74.9 Mb; p<0.0001), ISC-C (53–118 Mb; p<0.05) and ISC-D (64.6–74.9 Mb; p<0.0002) (Fig 1B). The strongest effect on survival was observed in mice carrying homozygous 129/129 alleles in ISC-B and ISC-D, with 8 week survival of 91.3% and 92.8%, respectively, compared to only 28.5% in heterozygous F1.*Scn1a*^+/-^ control mice (Fig 1B). ISC-D carried the smallest 129-derived segment (64.6–73.9 Mb) and refined the map interval to ~9 Mb (Fig 1A).

We next addressed whether the survival advantage conferred by 129 alleles in the ISC-D interval correlated with a decreased seizure burden. F1.*Scn1a*^+/-^offspring carrying either homozygous (129/129) or control heterozygous (129/B6) alleles in ISC-D were monitored from postnatal day 21 (P21) through P24 to determine the frequency of generalized tonic-clonic (GTC) seizures. *Scn1a*^*+/-*^ mice carrying homozygous (129/129) alleles in the ISC-D interval did not have a significantly reduced seizure frequency compared to heterozygous F1.*Scn1a*^+/-^ control mice (ISC-D: 0.024 ± 0.015 seizures/hr; F1 control: 0.022 ± 0.02 seizures/hr). Dissociation of the survival and seizure frequency phenotypes was somewhat surprising, and raised the question of whether seizure frequency and survival are correlated in F1.Scn1a^+/-^ mice. To address this question, we examined a separate phenotyping cohort and determined seizure counts for F1.*Scn1a*^+/-^ mice between P19 to P24. In F1.*Scn1a*^+/-^ mice experiencing seizures, total numbers of seizures were compared with survival throughout the monitoring period (Survivors: 7.95 ± 1.42 seizures; Lethals: 5.13 ± 1.55 seizures). This comparison revealed that high seizure frequency did not correlate with premature lethality in F1.*Scn1a*^+/-^ (Pearson r^2^ = 0.01, p>0.8111), consistent with our observations with ISC-D.

### Candidate Gene Identification by RNA-Seq

**Fig 2:**
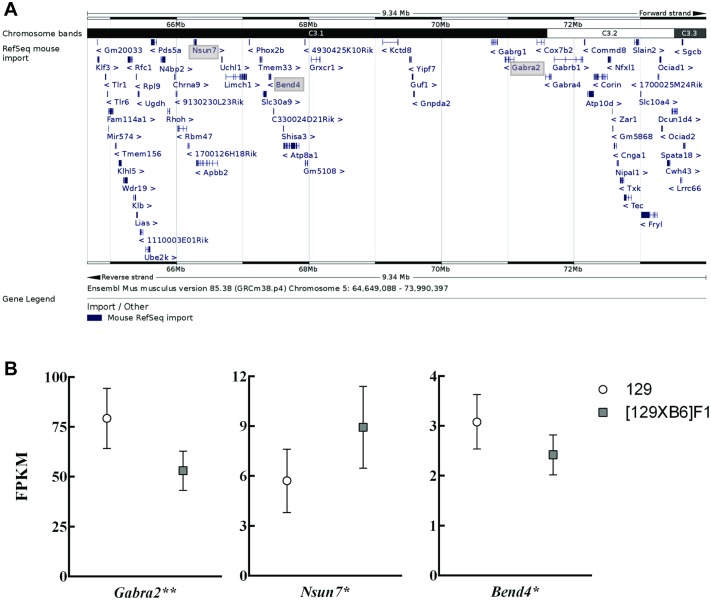
Evaluation of *Dsm1* Candidate Genes by RNA-Seq. A. The refined map interval is shown with mouse RefSeq gene annotations. The genes with strain-dependent differential expression are indicated by grey shading (Ensembl Mouse GRCm38.p4)[53]. B. Three genes within the *Dsm1* interval showed significant differences in brain expression between wildtype 129 and [129xB6]F1 mice: *Gabra2* (q < 0.0029**), *Nsun7* (q < 0.02*), and *Bend4* (q< 0.02*). Shown are RNA-Seq FPKM values and 95% confidence intervals calculated by String Tie. q-values are false discovery rate-adjusted p-values.

The *Dsm1* locus was narrowed to a 9 Mb region that contains 109 known and predicted genes, of which 40 are known to be expressed in the brain (Fig 2A). Within that interval, we identified four genes with non-synonymous coding sequence differences: *Phox2b*, *Tmem33*, *Slc30a9*, and *Fryl*. (Table 1). All resulting amino acid differences were predicted to be tolerated by SIFT [8].

**Table 1:** Coding sequence variants between C57BL/6J and 129S6/SvEvTac in *Dsm1* region.

Location*	Gene	Description	Variant	Consequence	SNP ID
Chr 5:67096280	*Phox2b*	paired-like homeobox 2b	G/A	Ala/Thr	-
Chr 5:67263874	*Tmem33*	Transmembrane protein 33/Shinc3	C/T	Ala/Val	rs29520321
Chr 5:67264487	*Tmem33*	Transmembrane protein 33/Shinc3	T/C	Phe/Leu	rs29510937
Chr 5:67324174	*Slc30a9*	solute carrier family 30 (zinc transporter), member 9/GAC63	T/C	Val/Ala	rs29548855
Chr 5:73064826	*Fryl*	FRY like transcription coactivator	G/A	Gly/Ser	rs33085471
Chr 5:73076182	*Fryl*	FRY like transcription coactivator	C/T	Ala/Val	rs29682128
Chr 5:73220556	*Fryl*	FRY like transcription coactivator	G/C	5' UTR variant	rs29683841

*Denotes location in GRCm38.p4 genome assembly.

To assess expression differences between 129 and [129xB6]F1 strains, we performed RNA-Seq on hippocampal RNA isolated from wildtype 129 and [129xB6]F1 mice at P24. Within the *Dsm1* interval, RNA-Seq identified three genes, *Nsun7*, *Bend4* and *Gabra2*, with significant expression differences between the 129 and [129xB6]F1 strains (Fig 2B, S1 Table). *Nsun7* is a putative RNA methyltransferase that when mutated, leads to reduced sperm motility in humans and mice [9,10]. *Bend4*, or BEN domain containing 4, is of unknown function. *Gabra2* encodes the GABA_A_ receptor α2 subunit, which has previously been associated with addiction and dependence [11–13]. Considering the importance of GABA signaling in epilepsy and reported dysfunction in GABAergic signaling in Dravet syndrome [14–16], *Gabra2* was identified as the top candidate modifier gene at *Dsm1*. Expression of *Gabra2* was significantly elevated in wildtype 129 mice compared to [129XB6]F1 mice (Fig 2B).

### Candidate Gene Expression Analysis

**Fig 3:**
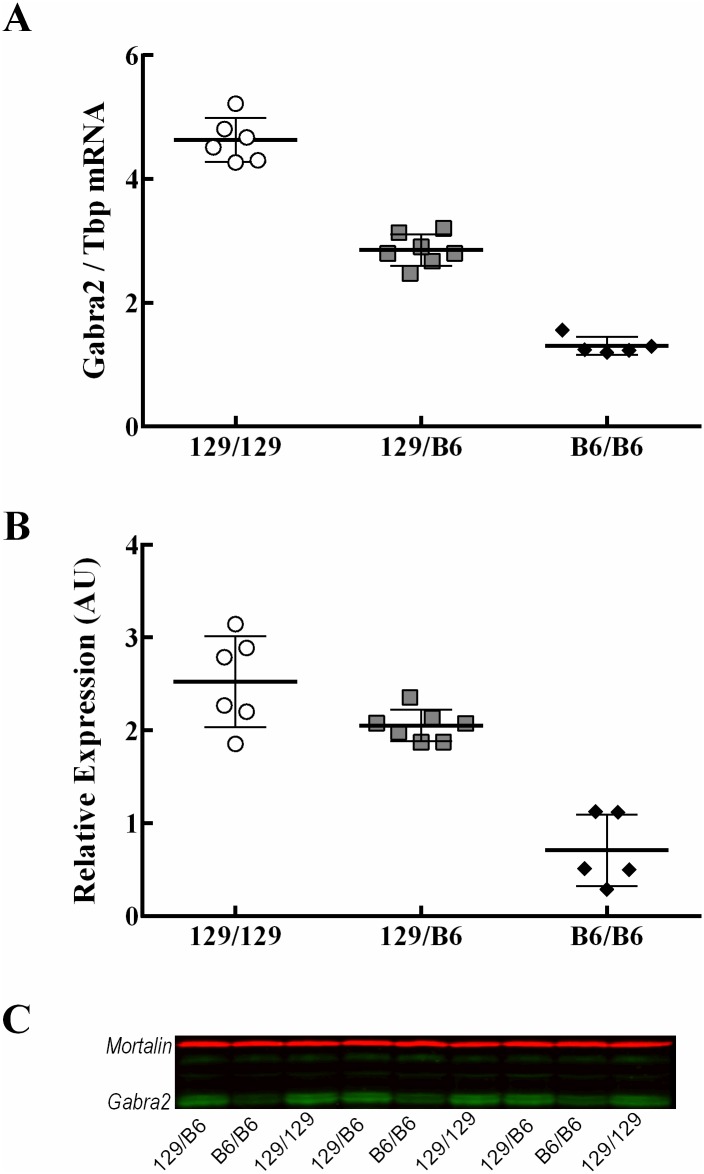
Allele specific *Gabra2* transcript and protein expression. A. Relative brain *Gabra2* transcript levels between mice carrying 129/129, 129/B6 or B6/B6 alleles in ISC-D were determined by ddRT-PCR and expressed are as a ratio of *Gabra2* to *Tbp*. Highest levels of *Gabra2* transcript are observed with homozygous 129/129 alleles, intermediate levels with heterozygosity, and lowest levels with B6 homozygosity. B and C. Brain GABRA2 protein expression for mice carrying 129/129, 129/B6 or B6/B6 alleles in ISC-D was assayed by immunoblotting using a rabbit polyclonal antibody for GABRA2 (Phosphosolutions, 822-GA2C; 1:2000) and a mouse monoclonal antibody for mortalin (NeuroMab 75–127; 1:1000) as a loading control. Relative levels were determined by densitometry and expressed as a ratio of GABRA2 to mortalin. Relative GABRA2 protein expression followed the same trend as *Gabra2* transcript. Scatter plot data points represent samples from individual mice (n = 5–6 biological replicates). Average values are depicted as a horizontal bar and error bars represent the SD. Statistical comparison between groups was made using ANOVA with Tukey’s post-hoc tests.

We further evaluated forebrain *Gabra2* transcript and protein expression in ISC-D mice carrying homozygous 129/129, heterozygous 129/B6 or homozygous B6/B6 alleles in *Dsm1*. We used digital droplet RT-PCR (ddRT-PCR) to evaluate transcript expression of *Gabra2*, as well as expression of other GABA_A_ receptor genes located within *Dsm1*, including *Gabra4*, *Gabrb1* and *Gabrg1*. *Gabra2* expression was significantly different between all genotypes (F_2,15_ = 206.4, p<0.0001). We observed the highest levels of *Gabra2* transcript with homozygosity for 129 alleles in *Dsm1*, intermediate levels with heterozygosity, and the lowest levels with B6 homozygosity (Fig 3A). Transcript expression of the other *Gabr* genes in the interval did not differ between genotypes (S1 Fig). Relative GABRA2 protein expression determined by immunoblotting correlated with the transcript levels, showing the highest expression with homozygous 129 alleles in *Dsm1*, intermediate with heterozygous alleles, and lowest with B6 homozygosity (Fig 3B and 3C). These results demonstrate that the level of *Gabra2* expression is regulated by alleles within the *Dsm1* interval, and that high expression of *Gabra2* is correlated with the protective allele for *Scn1a*^*+/-*^ survival.

### Pharmacological Modulation of *Gabra2*

**Fig 4:**
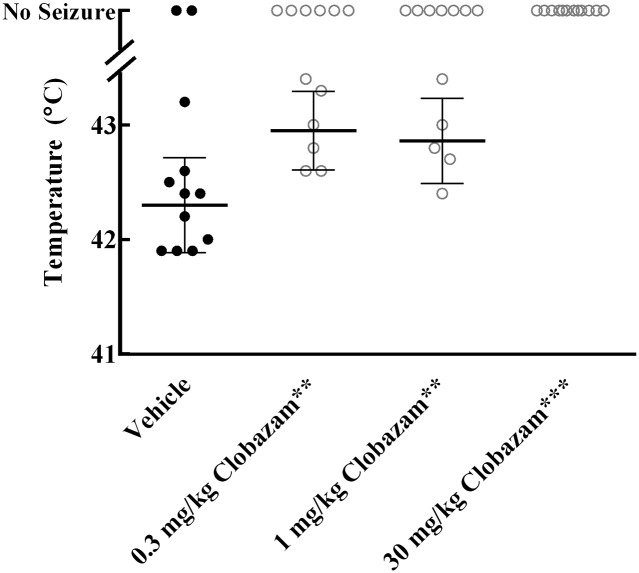
Pharmacological modulation of *Gabra2* by clobazam. F1.*Scn1a*^+/-^ mice underwent hyperthermia-induced seizure threshold testing after IP injections of vehicle or clobazam. The temperature at which generalized tonic-clonic seizure activity occurred is shown. Clobazam administration provided dose-dependent protection against hyperthermia-induced seizures. p values were determined by Mantel-Cox log-rank test (**p<0.01; ***p<0.001).

We used clobazam, an anticonvulsant drug that has preferential affinity for the GABRA2 subunit [17–19], as a pharmacological tool to further evaluate *Gabra2* as a candidate modifier gene. Both Dravet syndrome patients and F1.*Scn1a*^+/-^ Dravet mice experience seizures triggered by hyperthermia. We evaluated the effect of clobazam on survival and hyperthermia seizure threshold in F1.*Scn1a*^+/-^ mice. Beginning at P18, F1.*Scn1a*^+/-^ mice were fed either chow containing clobazam (320 mg per kg of chow; estimated dosage 40 mg/kg/day) or control chow, and survival was monitored to 30 days of age. This dose of clobazam, selected to achieve plasma concentrations within the human therapeutic range, was not sufficient to provide protection against survival. We used the hyperthermia-induced seizure assay to perform an acute dose-response study. Between P14–16, F1.*Scn1a*^+/-^ mice received an intraperitoneal injection of vehicle or clobazam (0.3, 1, or 30 mg/kg) prior to the induction of hyperthermia. Clobazam administration provided dose-dependent protection against hyperthermia-induced seizures (Fig 4). Clobazam dosed at 30 mg/kg provided complete protection against hyperthermia-induced seizures, while 83% of vehicle controls experienced GTC seizures with an average threshold temperature of 42.3°C (p < 0.0006). Clobazam doses of 0.3 and 1 mg/kg significantly increased GTC seizure thresholds to 43.0°C (p < 0.0022) and 42.9°C (p < 0.0079), respectively. To ensure plasma concentrations of clobazam reflected the human therapeutic range (0.1–0.4 μg/mL), plasma samples were assayed by HPLC. The commonly cited clobazam rodent dose of 30 mg/kg [20–22] resulted in an average plasma concentration that was 15 times higher (5.84 ug/mL) than the human therapeutic range, while the 1 mg/kg dose resulted in an average plasma concentration (0.16 ug/mL) within the range. Plasma concentrations were below the detection limit of the HPLC assay following the 0.3 mg/kg dose.

## Discussion

Candidate gene and expression analysis, along with pharmacological testing, identified *Gabra2* as a putative modifier gene that influences survival in the *Scn1a*^+/-^ Dravet mouse model. Considering the significance of GABAergic pathways in relation to epilepsy, strain-specific expression of *Gabra2* may differentially influence excitatory/inhibitory balance. Several GWAS studies have demonstrated that human *GABRA2* genetic variants are associated with alcohol dependence [23–27]. However, to date, no *GABRA2* genetic variants have been associated with human epilepsy. Altered *GABRA2* transcript and protein expression has observed in a number of rodent seizure models and patients with focal cortical dysplasia, tuberous sclerosis and temporal lobe epilepsy, suggesting a potential link between *GABRA2* expression and epilepsy [28–32]. There is evidence for variation in human cortical *GABRA2* expression that is associated with a trans eQTL on chromosome Xp11.4 [33]. Our mouse RNA-Seq and expression analysis demonstrated that low *Gabra2* expression associated with the B6 allele and correlated with reduced survival in the *Scn1a*^+/-^ model. Previous work has shown that in some mouse strains, *Gabra2* expression is strongly modulated by a *cis* eQTL that overlaps with *Dsm1* and a *trans* eQTL on chromosome 10 [34]. The difference in *Gabra2* expression was maintained when we examined transcript and protein levels in brain tissues from the ISC-D line, which contains 129 alleles only in the minimal interval and is B6 elsewhere in the genome. From this, we infer that *Gabra2* expression difference arises from a local eQTL effect within the *Dsm1* interval. This is supported by a recent study that demonstrated differential allele expression of *Gabra2* transcript in B6/129SF1 mice, with the 129 allele expressed at 2–3 times the level of the B6 allele [35]. Future work will focus on identifying the responsible regulatory variants within the *Gabra2* genomic region.

The failure of line ISC-D to reduce seizure frequency in F1.*Scn1a*^*+/-*^ mice was not surprising. As we report here, and have observed in our previous studies, high seizure counts do not correlate with premature death in F1.*Scn1a*^*+/-*^ mice. Therefore, we could exclude the survival improvement of the ISC-D line to be a result of altered seizure frequency. GABAergic neurons are found in several brain regions involved in the control of respiration and cardiac activity, and dysfunction in either pathway is believed to be primarily responsible for SUDEP [36–39]. Mice with a conditional *Scn1a*^*+/-*^ deletion in forebrain GABAergic interneurons experience SUDEP and cardiac dysfunctions similar to the Dravet *Scn1a*^*+/-*^ mouse model [40]. When *Scn1a*^*+/-*^ was conditionally deleted in the heart, the phenotype was not observed, demonstrating that SUDEP observed in *Scn1a*^*+/-*^ mice originates from the central nervous system [40]. Increased expression of *GABRA2* may exert a protective effect by altering autonomic control of cardiac and/or respiratory function and may explain the improved survival observed with 129/129 homozygosity in ISC-D. Additionally, GABA_A_ receptor activity plays a pivotal role in neuronal development and altered levels of *Gabra2* during development may alter network excitability [41].

Interestingly, clobazam, which has preferential affinity for the GABRA2 subunit, offers some therapeutic benefit in Dravet syndrome patients [17–19,42,43]. In our studies, threshold temperature for hyperthermia-induced seizures was elevated in *Scn1a*^+/-^ mice treated with supratherapeutic levels of clobazam, while lower doses did not provide significant protection from hyperthermia-induced seizures or premature death. The 30mg/kg clobazam dose provided complete protection against hyperthermia-induced seizures, but resulted in plasma levels far above the human therapeutic range, highlighting the necessity of determining drug concentrations in similar studies. Frequently, Dravet syndrome patients are prescribed stiripentol as an add-on treatment to clobazam. In a randomized placebo-controlled study in children with Dravet syndrome, 71% of patients treated with a combination of clobazam and stiripentol experienced a reduction in seizure frequency [44]. Although the mechanism of action for the anticonvulsant benefit of stiripentol is yet to be determined, stiripentol has been identified as an inhibitor of CYP450s, specifically CYP3A4 and more potently CYP2C19, both important for the metabolism of clobazam. *In vivo*, a significant increase in the plasma concentrations of both clobazam and its active metabolite *N*-desmethylclobazam has been shown with concomitant stiripentol treatment [45], suggesting that elevated levels of clobazam may account for some of the therapeutic benefit.

Identification of *Gabra2* as a putative modifier gene at the *Dsm1* locus furthers our understanding of the genetic basis of Dravet syndrome. This provides information that may help predict the risk of SUDEP and the clinical course of epilepsy due to a sodium channel mutation. Furthermore, modifier genes may suggest new targets for the improved treatment of epilepsy.

## Methods

### Ethics Statement

All studies were approved by the Vanderbilt University [M/06/499 JAK] and Northwestern University Animal Care and Use Committees [IS00000539 JAK] in accordance with the National Institutes of Health Guide for the Care and Use of Laboratory Animals. Principles outlined in the ARRIVE (Animal Research: Reporting of in vivo Experiments) guideline and Basel declaration (including the 3R concept) were considered when planning experiments.

### Mice

*Scn1a*^*tm1Kea*^ mice, with deletion of the first coding exon, were generated by homologous recombination in TL1 ES cells (129S6/SvEvTac) as previously described [7]. The 129.*Scn1a*^*+/-*^ line has been maintained by continuous backcrossing to 129S6/SvEvTac (129). To generate mice for hyperthermia-induced seizure experiments, 129.*Scn1a*^*+/-*^ mice were crossed with C57BL/6J (B6) resulting in [129XB6]F1.*Scn1a*^*+/-*^ (F1.*Scn1a*^*+/-*^) mice. Mice were maintained in a Specific Pathogen Free (SPF) barrier facility with access to food and water *ad libitum*.

### Generation of Interval Specific Congenic (ISC) lines

We generated five ISC lines carrying 129-derived chromosome 5 segments on a B6 background. [129 x B6]F1 progeny were continually backcrossed to B6 to generate congenic lines. Genotyping for chromosome 5 markers was performed at each generation and animals retaining 129 alleles in the *Dsm1* interval were propagated. Whole genome and selective genotyping was performed at generations N2–N7 to select breeders with the lowest percentage of 129 alleles in the rest of the genome. All ISC lines were backcrossed to B6 for ≥N6 generations prior to experiments.

### Phenotyping

*Dsm1* ISC females were crossed with heterozygous *Scn1a*^*+/-*^ males to generate (B6.ISC x 129.*Scn1a*^*+/-*^)F1 offspring carrying heterozygous (129/B6) or homozygous (129/129) alleles in *Dsm1*. Phenotyping was performed similarly to our low-resolution mapping study [7]. Mice were ear-tagged at P12–14. At P21, mice were weaned into holding cages containing four to five mice of the same age and sex. Wild-type littermates were included in all holding cages. Survival was monitored to 8 weeks of age. During that time, all mice were monitored daily for general health and any mouse visibly unhealthy (e.g. underweight, dehydrated, poorly groomed, or immobile) was euthanized and excluded from the study. The focus of the study was sudden and unexpected death in the *Scn1a*^*+/−*^ mice occurring in otherwise healthy appearing animals.

We used continuous video monitoring to determine GTC seizure frequency in *Scn1a*^*+/-*^ mice with homozygous or heterozygous alleles in ISC-D mice. At P20, mice were placed in a recording chamber and video was captured using a Day/Night camera (Samsung SCB5003) equipped with an infrared lens (Tamron 13FG04IRSQ) and saved to a DVR (Samsung SRD-876D). During recording, mice had *ad libitum* access to food and water, and were maintained on a 14:10 light-dark cycle. Video records were analyzed offline by an observer blinded to genotype. We validated use of video capture for seizure evaluation in *Scn1a*^+/-^ mice in a preliminary study. Behavioral GTC seizures were correlated with electroencephalographic seizures using video-electroencephalography (EEG) monitoring as previously described. On P16, F1.*Scn1a*^+/-^ mice were implanted with prefabricated headmounts (Pinnacle Technology, Inc., Lawrence, KS, USA) and mice were allowed to recover for >72 hours. Continuous video-EEG data monitoring was performed from P20-P26. Data were acquired and analyzed with Sirenia software (Pinnacle Technology, Inc.). Electrographic GTC seizure activity was scored manually using video-EEG data. Separately, behavioral GTC seizures were counted using only the video record. There was perfect agreement between the video-EEG and video-only results (κ = 1.0; n = 25 mice; 39 seizures), validating video recording as a reliable method for measuring GTC seizure frequency in *Scn1a*^*+/-*^ mice.

### Genotyping

DNA was prepared from tail biopsies using the Gentra Puregene Mouse Tail Kit according to the manufacturer’s instructions (Qiagen, Valencia, CA, USA). *Scn1a* genotype was determined by multiplex PCR as previously described [7]. Microsatellite genotyping was performed by analysis of PCR products on 7% denaturing polyacrylamide gels stained with ethidium bromide.

### RNA-Seq

Hippocampi were dissected from 129 and [129 x B6]F1 mice at P24. Primary pools were created with tissue from both male (n = 2) and female (n = 2). Total RNA was isolated using TRIzol reagent according to the manufacturer’s instructions (Life Technologies). Following RNA isolation, RNA integrity was assessed and all samples had a RIN of ≥ 7.7. For each strain, three superpooled biological replicates were generated by combining total RNAs from 3–4 primary pools (n = 12–16 mice/superpool). RNA integrity was assessed on the superpool samples and all samples had a RIN of ≥ 8.1.

Samples were processed for RNA-Seq using the TruSeq RNA Library Preparation Kit v2 (Illumina, San Diego, CA, USA). Samples were sequenced on an Illumina HiSeq 4000 at BGI (Hong Kong, China). Three multiplexed lanes of 50-bp single-end sequencing resulted in almost 167 million mapped reads. Base calling and filtering of sequence reads were performed with the Illumina pipeline [46]. Bioinformatic analysis was performed using Tuxedo Tools on the GALAXY platform [47–49]. *Tophat2* aligned reads with *Bowtie2* and identified splice junctions [50]. StringTie assembled alignments into transcripts [51]. *Cuffmerge* and *Cuffcompare* combined biological replicate transcript files and assigned reference annotations to transcripts [52]. *Cuffdiff* provided significance values for expressed genes and transcripts [52].

### ddRT-PCR

Forebrain RNA was extracted from P24 ISC-D mice carrying homozygous B6, homozygous 129, or heterozygous alleles. Total RNA was isolated using TRIzol reagent according to the manufacturer’s instructions. First-strand cDNA was synthesized from 2 micrograms of RNA using oligo(dT) primer and Superscript IV reverse transcriptase according to the manufacturer’s instructions (Life Technologies). First-strand cDNA samples were diluted 1:10 and 5 μl was used as template. Quantitative digital droplet PCR (ddPCR) was performed using ddPCR Supermix for Probes (No dUTP) (Bio-Rad, Hercules, CA, USA) and TaqMan Gene Expression Assays (Life Technologies) for mouse *Gabra2* (FAM-MGB-Mm00433435_m1), *Gabra4* (FAM-MGB-Mm00802631_m1), *Gabrb1* (FAM-MGB-Mm00433461_m1), *Gabrg1* (FAM-MGB-Mm00439047_m1) and *Tbp* (VIC-MGB-Mm00446971_m1). Reactions were partitioned into 20,000 droplets (1 nL each) in a QX200 droplet generator (Bio-Rad). Thermocycling conditions were 95°C for 10 minutes, then 40 cycles of 95°C for 15 seconds and 60°C for 1 minute (ramp rate of 2°C/sec) and a final inactivation step of 98°C for 10 minutes. Following amplification, droplets were analyzed with a QX200 droplet reader with QuantaSoft v1.6.6.0320 software (Bio-Rad). All assays lacked detectable signal in no-RT and no template controls. Relative transcript levels were expressed as a ratio of the gene of interest concentration to *Tbp* concentration. Statistical comparison between groups was made using ANOVA with Tukey’s post-hoc tests. Data are presented as mean ± SD of 5–6 biological replicates.

### Immunoblotting

Forebrain membrane proteins were isolated from P24 ISC-D mice carrying homozygous B6, homozygous 129, or heterozygous alleles. Membrane fractions (50 μg/lane) were separated on a 7.5% SDS-PAGE gel and transferred to nitrocellulose membranes. Immunoblots were probed for GABRA2 using a rabbit polyclonal antibody (Phosphosolutions, 822-GA2C) and for Mortalin using a mouse monoclonal antibody (NeuroMab, 75–127). Alexa-conjugated fluorescent secondary antibodies (Jackson ImmunoResearch) were used to detect bound primary antibody using an Odyssey imaging system (Licor).

### Hyperthermia Induced Seizures

F1.*Scn1a*^*+/-*^ mice between P14 to P16 received an intraperitoneal injection of 0, 0.3, 1, or 30 mg/kg doses of clobazam solubilized in vegetable oil (10 ml/kg volume). After 15 minutes, each mouse was placed in a cylindrical container and fitted with a RET-3 rectal probe (Physitemp) connected to a heat lamp via a temperature controller (TCAT-2DF (Physitemp) reconfigured with a Partlow 1160+ controller). At 20 minutes post-injection, mouse body temperature was elevated by 0.5°C every two minutes until a maximum of 42.5°C was reached. When a GTC seizure occurred (rearing and falling with forelimb clonus), mice were removed from the container and temperature was recorded. If after 3 minutes at 42.5°C no seizure occurred, the mouse was removed and recorded as a seizure-free.

### Clobazam Assay by HPLC

Mice that underwent hyperthermia-induced seizure threshold testing were immediately removed from the assay and anesthetized with isoflurane. Cardiac puncture was performed and whole blood was collected in heparin microtainers (BD365965). Plasma was isolated by centrifugation (9000 x g for 10 minutes at 4°C). Plasma samples (50–100 μL) were spiked with 5 μg/mL prazepam (250 μg/mL in methanol) as an internal standard and vortexed well. Extraction of clobazam and prazepam was achieved by vortex-mixing with diethyl ether (4x volume). The organic layer was isolated following centrifugation at 2000 rpm for 5 minutes and was evaporated by heating at 75°C. The residue was reconstituted in acetonitrile:20 mM potassium phosphate buffer, pH 7 (42:58, v/v).

Clobazam was detected in plasma samples using a HPLC 9 Flexar Binary LC Pump Platform and Flexar UV/Vis Detector (Perkin Elmer, Waltham, MA) with a Polaris C-18A 5 μm column (4.6 x 100 mm; Agilent Technologies, Santa Clara, CA). The mobile phase consisted of acetonitrile and 20 mM potassium phosphate, pH7 (42:58, v/v) with a flow rate of 1.5 mL/min and detection at a wavelength of 228 nm. Quantitative analysis clobazam was performed using a calibration curve (0.05–1.0 μg/mL) by spiking clobazam into blank mouse plasma and preparing as described above.

## Supporting Information

S1 Fig *GABR* subunit expression analysis. Brain *Gabr* transcript levels were compared between mice carrying 129/129, 129/B6 or B6/B6 alleles in ISC-D as determined by ddRT-PCR. Transcript levels are expressed relative to *Tbp* for *Gabra4* (**A**), *Gabrb1* (**B**), and *Gabrg1* (**C**). For all three genes, no difference in transcript levels were observed between homozygous 129/129, heterozygous 129/B6 or homozygous B6/B6. Scatter plot data points represent samples from individual mice (n = 5–7 biological replicates). Average values are depicted as a horizontal bar and error bars represent the SD. Statistical comparison between groups was made using ANOVA with Tukey’s post-hoc tests.
(TIF)

S1 Table Cuffdiff output for *Dsm1* region on mouse chromosome 5. (XLSX)
